# Assessing the effect of multibracket appliance treatment on tooth color by using electronic measurement

**DOI:** 10.1186/s13005-018-0174-4

**Published:** 2018-10-22

**Authors:** Anja Ratzmann, Christan Schwahn, Anja Treichel, Andreas Faltermeier, Alexander Welk

**Affiliations:** 1 Department of Orthodontics and Department of Dental Propaedeutics/Community Dentistry, Dental School, University Medicine, Walther-Rathenau Straße 42a, 17475 Greifswald, Germany; 2 Department of Prosthetic Dentistry, Gerodontology and Biomaterials, University of Greifswald, Fleischmannstraße 42, 17475 Greifswald, Germany; 3 Private Dental Office, Bahnhofstraße 4, 18581 Putbus, Germany; 4 Department of Orthodontics, Dental School, University Medicine, Franz-Josef-Strauß-Allee 11, 93053 Regensburg, Germany; 5 Department of Restorative Dentistry, Periodontology, Endodontology, Preventive and Pediatric Dentistry, Dental School, University Medicine, Walther-Rathenau Straße 42a, 17475 Greifswald, Germany

## Abstract

**Background:**

The purpose of this study was to investigate how tooth color is affected by multibracket appliance (MBA) treatment.

**Methods:**

The color of teeth #14 to #24 of 15 patients with MBA was measured on body and gingival tooth segments using the spectrophotometer Shade Inspector™. Colors of both segments were recorded before start of MBA treatment (baseline T_0_), end of MBA treatment (T_1_; 2 years ±0.3), and 3 months after T_1_ (T_2_). A 2D color system and a 3D system served as reference systems.

Multilevel models were used to analyze color change within segments and to compare the difference in color change between segments (treatment effect).

**Results:**

2D system. Changes within tooth segments from T_0_ to T_2_ were at worst 2.0 units (ΔE in the gingival segment), which is less than the threshold of 2.7 units for a clinically meaningful difference. Confidence intervals for the treatment effect indicated no clinically important differences in color change between body and gingival segments.

3D system. Changes within tooth segments from T_0_ to T_2_ were at worst 2.3 units (ΔE in the body segment), which is less than the threshold of 2.7 units for a clinically meaningful difference. Confidence intervals for the treatment effect indicated no clinically important differences in color change between body and gingival segments.

Thus, MBA treatment did not lead to clinically relevant changes in tooth color.

**Conclusion:**

Within the limitation of this study the MBA treatment can be seen as a safe method with respect to tooth color.

## Background

Changes in tooth color may be caused by several factors, for instance, by extrinsic (external) and intrinsic (internal) discolorations, or by aging [1]. Further causes of color changes are dental treatments, including bleaching or restorative therapy [2]. In addition, tooth color can be changed by the acid-etching process used for bonding orthodontic brackets [3]. Formation of white spots and irreversible penetration of resin tags that remain in the enamel as the two main causes have been reported [4–7]. Therefore, multibracket treatment (MBA) may be associated with enamel discoloration due to changes in the enamel by tooth cleaning, enamel conditioning procedures (etching), and the debonding and subsequent polishing processes [8, 9].

Association between tooth color changes due to bonding and debonding procedure and multibracket treatment (MBA) is discussed controversial. Some studies [4, 10, 11] have shown that enamel color variables were significantly affected by bonding and debonding procedures, other investigations [3, 12–14] did not find clinically important influence of this procedures on the enamel discolorations.

The purpose of this in vivo study was to investigate how tooth color is affected by multibracket appliance (MBA) treatment, especially whether: (1) the change in tooth color during MBA treatment is clinically important; (2) the color change differs by bracket (body) and non-bracket (gingival) tooth segments; and (3) the change is substantially the same for the conventionally used 2D system and the scientifically favorable 3D system.

## Methods

### Subjects and clinical examination procedure

**Fig. 1:**
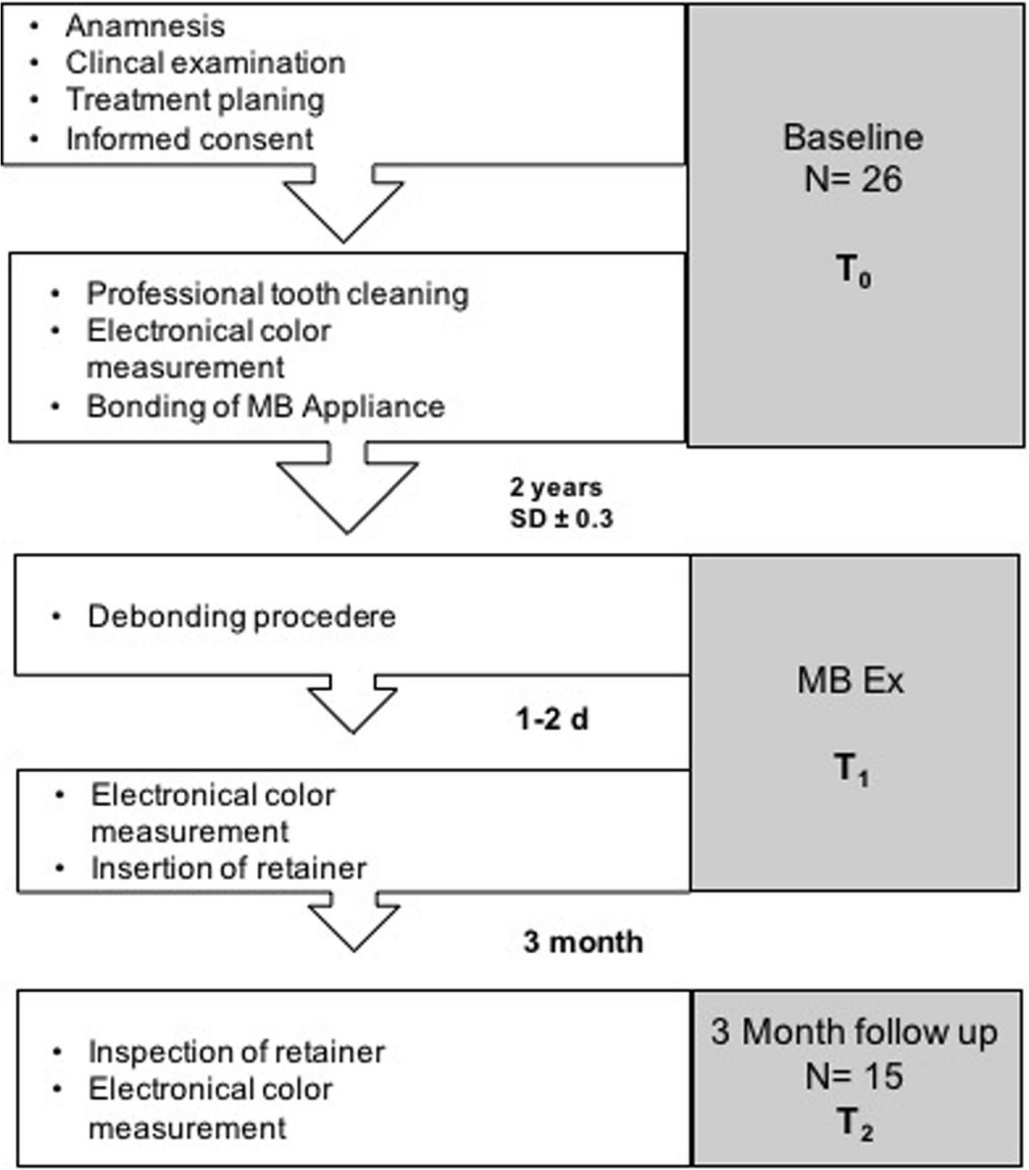
Consort Flow Diagram

**Fig. 2:**
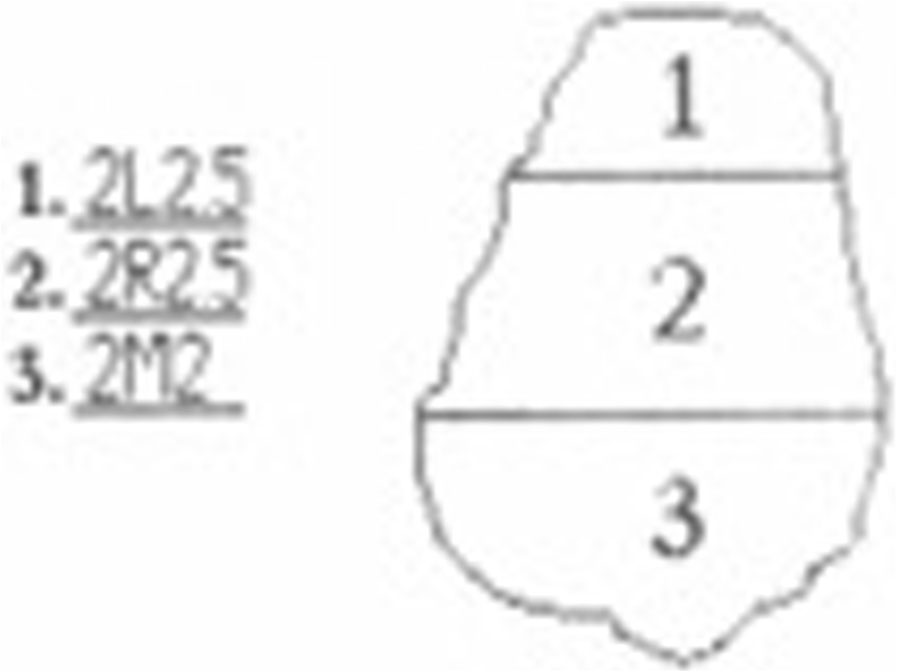
Measuring report by Shade Inspector™

**Fig. 3:**
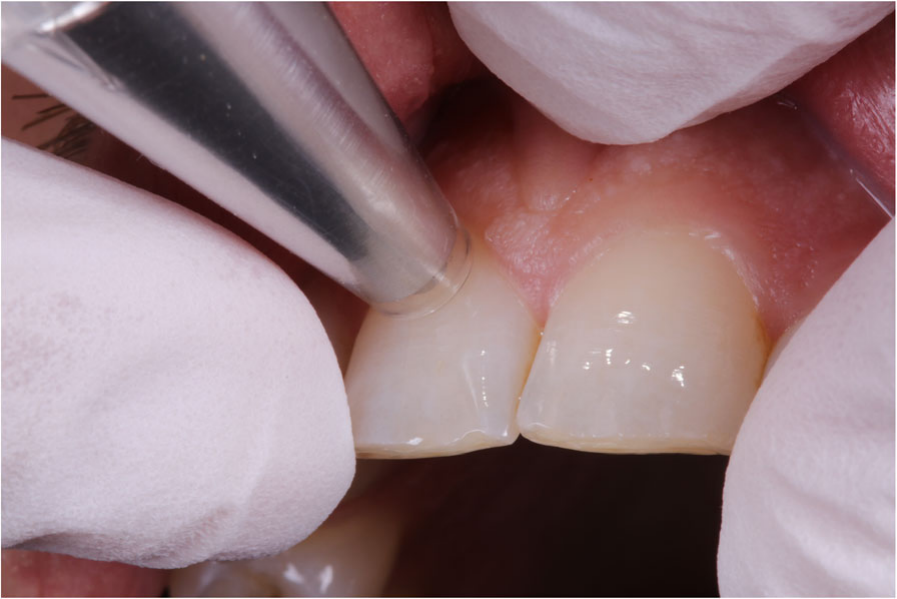
Shade Inspector™ - Measurement of the gingival segment (S_1_)

All subjects expecting MBA treatment were regular patients of the orthodontic department and participated on a voluntary basis. All measurements were performed during regular visits. All procedures performed in this study were in accordance with the ethical standards of the institutional research committee Ärztekammer Mecklenburg-Vorpommern (Reg. Nr.III UV 15/08). Informed consent was obtained from the patients and parents before start of the study. Initially, 26 patients were included. The inclusion criteria were good oral hygiene, non-carious and restoration-free permanent teeth, and no white spots. The multibracket appliances had been present in situ for 2.0 (SD ± 0.3) years (individual study period of each patient). The entire period of study data collection lasted from 2005 to 2009. Time points of measurements were start of MBA treatment (baseline - T_0_), end of MBA treatment (2 years SD ± 0.3 - T_1_), and 3-month after end of MBA treatment (T_2_) (Fig. 1). The complete clinical procedure was performed by an experienced orthodontist under standardized conditions (color neutral such as same room and light conditions, patient was covered by a drape, tooth surfaces were always saliva-wet) according to the standardized bonding protocol of the orthodontic department. Enamel was etched with 35% orthoposphoric acid (Scotchbond, 3 M Unitek) for 10 s, rinsed with air-water spray for 20 s and dried for 10 s. Transbond XT™ Ligth Cure Primer (3 M, Unitek) was used in conjunction with Transbond XT™ Ligth Cure Adhesive (3 M Unitek) according to the manufacturer’s instructions for bonding Mini-Mono – .022 Roth Technique Stainless Steel Brackets (Forestadent, Germany). After that the bracket was pressed firmly on the enamel surface and the excess adhesive resin was removed with a probe. Light curing was performed with LED source Starlight Pro (Mectron, Germany) for 10 s. For study purposes, the protocol was slightly modified by the additional advice “avoiding etching of the gingival segment”. Each tooth was categorized into the gingival (S_1_), the body (S_2_), and the incisal (S_3_) segment (Fig. 2). For standardization of the measurements, we used the facial axis point (FA point) for placing the bracket determined with a Dental Bracket Placement Gauge accordingly the MBT™-technique for the middle segment S_2_ and for gingival segment S_1_ we placed the tip of the measuring probe perpendicularly 1 mm above of the middle point of the gingival line of the corresponding tooth (Fig. 3). The probe was moved slightly around the defined measurement points measuring automatically four times giving an overall value of these measurements at the end. The incisal segment S_3_ was not included into analysis because of its transparency. All measurements were performed by a calibrated examiner from a pilot study [15].

During the entire study period we lost 11 patients. Drop out reasons were lack of oral hygiene with breakup of fixed orthodontic treatment, move, repeated schedule failure and withdrawal of informed consent.

### Electronic color measurement

Tooth color was measured electronically with the spectrophotometer Shade Inspector™ (Schuetz Dental, Rosbach, Germany- presently not available). The tooth color measuring device operates independently of light on the principle of spectral photometry. For color determination, the color data of the test specimen are compared with manufacturer-furnished color rings. The tested spectrophotometer is calibrated with a factory-provided selection of industrially fabricated color reference scale VITAPAN Classical® and VITA 3D-Master® by the company (*Schuetz Dental, Rosbach, Germany).* In the present study, the color references VITAPAN Classical® and VITA SYSTEM 3D-Master® were selected from the device software. The *VITAPAN® Classical* Color System has a two-dimensional structure that enables the description of *hue* (category A to D) and *lightness* including *chroma* (group 1 to 4) [16, 17]. It serves as standard shade guide for visual color assessment in dental praxis. The *VITA 3D-Master®* Color System has a three-dimensional structure that enables the separate description of *lightness* (1 to 5 and 0 for bleaching), *chroma* (1 to 3, including half points), and *hue* (M, L, R) [18]. It was developed to obtain a method for systematic and ordered color determination and a better hit rate. The examiners were provided with device operating instructions to ensure observance of the manufacturer’s specifications and calibrated in a pilot study [15]. Within a 1 mm measurement range diameter, the probe measures 26 standard colors and three bleaching colors from the VITA 3D–Master® color ring as well as 16 standard colors and 48 intermediate colors (calculated) from the VITA Classical® color ring. The measuring probe was protected by a detachable hygiene cap. During the measurements the probe was placed vertically to the tooth surface (Fig. 3).

### Statistical methods

As the 3D-system (*VITA 3D-Master*) is “a more ordered shade guide” than the 2D-system (*VITAPAN*® *Classical*) [16], we considered the 3D-system as the primary outcome [19, 20].

Besides *lightness* and *chroma*, we analyzed color distributions in terms of L* (CIE lightness) and C*_ab_ (CIE chroma) after having assigned *VITA 3D-Master*® shades to values given in Table 1 in Ahn et al. [21] via data analysis syntax. Additionally to L*, values for a* and b* were calculated from values of C*_ab_ and *h* degrees as given in Ahn et al. and were then used to calculate *ΔE* (defined [22][as square root of [(ΔL*)^2^ + (Δa*)^2^ + (Δb*)^2^]). For example, the change from 1 M2 to 2 L2.5 was calculated in two steps. First, a* and b* values were calculated (a*_1M2_ = 8.7*cos(89.4*2*π/360) = 0.09; b*_1M2_ = 8.7*sin(89.4*2*π/360)); then the square root of [(65.0–61.3)^2^ + (0.09–0.82)^2^ + (8.70–13.5)^2^] = 6.1 was calculated, which can also be found in Table III in Ahn et al. [21]. Because *ΔE* is restricted to non-negative values, we computed the distance of each shade to 0 M1 additionally, denoted by d(0 M1). A positive change in d(0 M1) indicates a darker or stronger color; a negative change indicates a lighter or purer color.

**Table 1:** Description of color distributions on tooth level (*n* = 120)

	Time point	Gingival (S_1_)			Body (S_2_)			*P* value
		Mean	Gmd	Median (1st – 3rd quartile)	Mean	Gmd	Median (1st – 3rd quartile)	
3D system								
Lightness^a^	T_0_	1.93	0.72	2 (1–2)	1.84	0.75	2 (1–2)	0.170
	T_1_	2.02	0.83	2 (2–2)	1.96	0.94	2 (1–2)	
	T_2_	2.06	0.73	2 (2–2)	1.98	0.73	2 (2–2)	
Chroma^b^	T_0_	2.45	0.40	2.5 (2.0–2.5)	2.38	0.35	2.5 (2.0–2.5)	0.018
	T_1_	2.48	0.39	2.5 (2.0–2.5)	2.39	0.38	2.5 (2.0–2.5)	
	T_2_	2.54	0.39	2.5 (2.5–3.0)	2.48	0.39	2.5 (2.0–2.5)	
L*	T_0_	61.8	2.6	61.7 (61.3–65.0)	62.0	2.7	61.8 (61.3–65.0)	0.388
	T_1_	61.5	3.0	61.6 (61.3–61.8)	61.6	3.3	61.6 (61.3–65.0)	
	T_2_	61.4	2.5	61.6 (61.3–61.8)	61.6	2.5	61.6 (61.3–61.8)	
C*_ab_	T_0_	12.1	2.1	11.8 (8.7–14.3)	11.7	2.6	11.8 (8.7–13.5)	0.042
	T_1_	12.3	2.0	13.5 (10.1–14.3)	11.9	2.1	12.6 (8.7–14.3)	
	T_2_	12.7	1.7	13.5 (11.8–14.3)	12.2	1.9	11.8 (10.1–14.3)	
ΔE	T_0_ – T_1_	2.10	2.57	0.90 (0.00–4.22)	2.46	2.82	1.88 (0.00–4.42)	
	T_1_ – T_2_	2.15	2.54	1.88 (0.00–4.36)	2.35	2.50	1.88 (0.00–4.36)	
	T_0_ – T_2_	1.80	2.21	0.90 (0.00–3.48)	1.86	2.17	1.01 (0.00–3.60)	
d(OM1)	T_0_	13.9	3.8	13.7 (9.2–15.8)	13.5	3.9	13.7 (9.2–15.4)	0.038
	T_1_	14.3	4.1	15.3 (12.5–15.8)	13.9	4.5	15.3 (9.2–15.8)	
	T_2_	14.6	3.7	15.3 (13.7–15.8)	14.1	3.8	14.5 (12.5–15.8)	
2D system^c^								
L*	T_0_	58.1	2.1	58.4 (56.8–59.7)	58.4	2.1	58.4 (57.1–59.7)	0.004
	T_1_	57.8	2.4	58.4 (55.8–59.7)	58.1	2.4	58.4 (57.1–59.7)	
	T_2_	57.7	2.3	57.1 (55.8–59.7)	58.1	2.0	58.4 (57.1–59.7)	
C*_ab_	T_0_	12.4	2.1	12.3 (11.0–13.6)	12.2	2.1	12.3 (11.0–13.6)	0.031
	T_1_	12.7	2.4	12.3 (11.0–14.9)	12.4	2.6	12.3 (11.0–13.6)	
	T_2_	12.9	2.0	13.6 (11.0–14.9)	12.5	2.3	12.3 (11.0–13.6)	
ΔE	T_0_ – T_1_	1.63	1.79	1.81 (0.00–1.87)	1.74	1.78	1.87 (0.00–1.87)	
	T_1_ – T_2_	1.45	1.65	1.81 (0.00–1.87)	1.65	1.80	1.87 (0.00–1.91)	
	T_0_ – T_2_	1.61	1.62	1.87 (0.00–1.87)	1.39	1.42	1.84 (0.00–1.87)	

^a^3D lightness values were assessed on a six-point integer scale from 0 to 5

^b^3D chroma values were assessed on a three-point scale from 1 to 3 at half points

^c^2D second shade designation numbers were assessed on a five-point scale at quarter points

Gmd denotes Gini’s mean difference (see statistical methods)

In the 2D-system, the shade group B is ordered by C*_ab_ (CIE chroma), but not by L* (CIE lightness); for the latter B2 > B1 > B4 > B3) [16]. Therefore, we analyzed color distributions only in terms of L*, C*_ab_, and ΔE after having assigned *VITAPAN*® *Classical* shades to values given in the D_65_ columns of Table I in *Park* et al. [16] as described for the 3D system. Because the second shade designation numbers of the 2D-system were assessed on a five-point scale at quarter points, extrapolation to five and interpolation to quarter points were applied.

In the 2D-system, the shade group B is ordered by C*_ab_ (CIE chroma), but not by L* (CIE lightness); for the latter B2 > B1 > B4 > B3) [16]. Therefore, we analyzed color distributions only in terms of L*, C*_ab_, and ΔE after having assigned *VITAPAN*® *Classical* shades to values given in the D_65_ columns of Table I in *Park* et al. [16] as described for the 3D system. Because the second shade designation numbers of the 2D-system were assessed on a five-point scale at quarter points, extrapolation to five and interpolation to quarter points were applied.

As the *American Statistical Association* [23] recommends to avoid over-reliance on *p*-values, we estimated and interpreted confidence intervals [24]. Treatment effects were corrected for tooth level and subject level by using multilevel modeling [25], and adjusted for tooth type and quadrant. The group difference in change from baseline was calculated in order to estimate treatment effects. Originally, a difference in shade ≥3.7 CIELAB units had been prespecified as clinically meaningful both for changes within groups and treatment effects [16] which was revised to ≥2.7 [26]. The treatment group difference in change (change in S_1_ versus change in S_2_) was estimated by linear multilevel models with Kenward-Roger correction for small samples [27] via the procedure “mixed” by Stata software, release 14.2 (Stata Corporation, College Station, TX, USA); changes within groups were computed afterwards using the command “margin”. The relative treatment effect of the difference in change was estimated by ordinal logistic multilevel models via Stata’s procedure “meologit”. Odds ratios in the ordinal logistic regression can be interpreted as those in the binary logistic regression whatever the cutoff point of the ordinal outcome is [28]. Box plots and descriptive statistics, including quantiles and Gini’s mean difference (Gmd) as a robust measure of dispersion [28], were generated using R, release 3.3.3 (R Core Team (2017). R: A Language and Environment for Statistical Computing. R Foundation for Statistical Computing. Vienna, Austria. https://www.r-project.org), especially the “ggplot2” package [29].

## Results

### Subjects, teeth, and observations

The initial study sample consisted of 26 consecutive patients. Eleven patients were excluded from the study for different reasons, including lack of oral hygiene, decalcification, or relocation. The multibracket appliances had been present in situ for 2.0 years (SD ± 0.3). At the end of MBA treatment, data for tooth color of 120 teeth of the upper jaw (#14 to #24) of 12 female and 3 males were available, resulting in a total of 720 observations for each color system (120 teeth, 2 tooth segments, 3 time points). All patients were Caucasian, aged 11 to 18 years.

### Measurements results

#### 2D-system

**Fig. 4:**
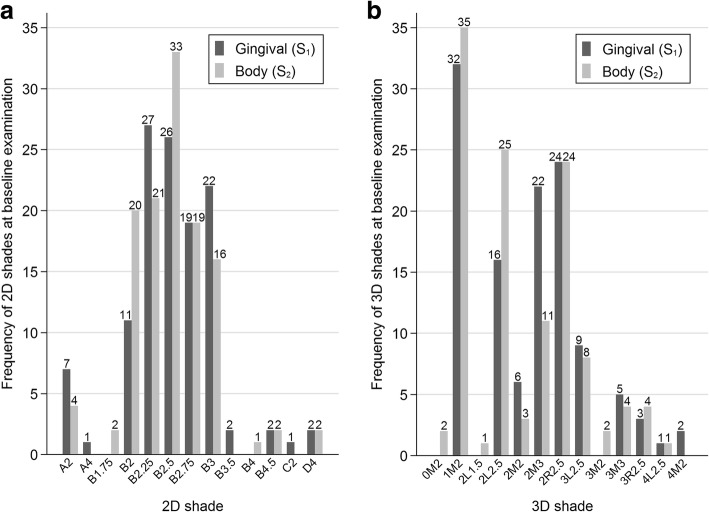
**a**, **b** Frequencies of 2D and 3D shades in gingival and body segments of 120 teeth at baseline

**Fig. 5:**
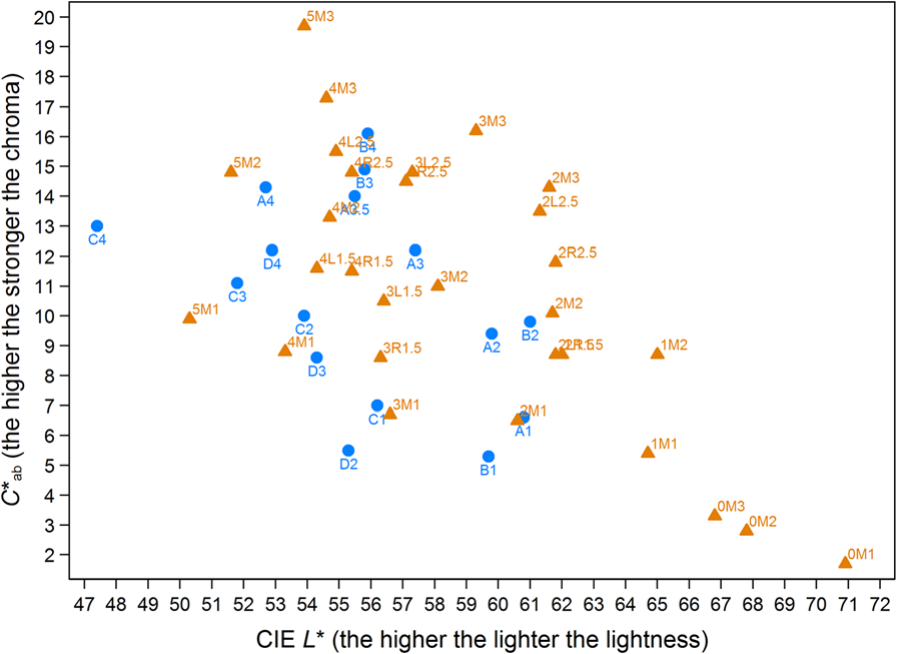
Scatterplot of CIE L* and C*_ab_ values for 2D shades (blue) and 3D shades (orange)

At baseline, 13 different shades were measured (Fig. 4a). Five shades with a frequency greater than 30 occurred: B2, B2.25, B2.5, B2.75 and B3 (Fig. 4a). Coordinates (CIE L*, a*, b*) of quarter points for the second shade designation number were interpolated to (61.0, 59.7, 58.4, 57.1, and 55.8) for L* of B2, B2.25, B2.5, B2.75 and B3, respectively, and to (9.8, 11.1, 12.4, 13.6, and 14.9) for C*_ab_ of B2, B2.25, B2.5, B2.75 and B3, respectively (Fig. 5). Note that B2.25, B2.5, and B2.75 lie in a space not well covered by the 3D-system (Fig. 5). Gingival segments were darker (L*) and stronger (C*_ab_) than body segments (*P* = 0.004 and *P* = 0.031, respectively; Table 1).

**Fig. 6:**
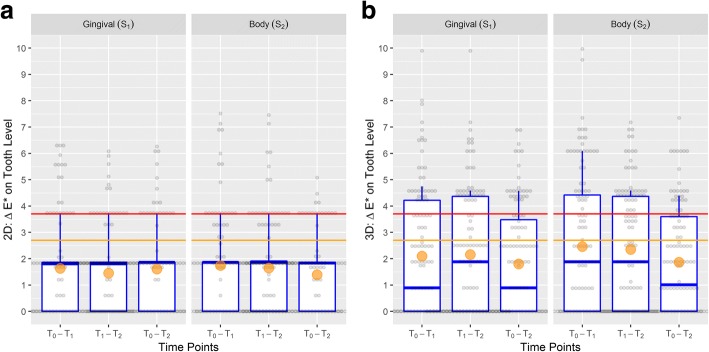
**a**, **b** Box plots showing the distribution of ΔE for the 2D-system (**a**; left) and the 3D-system (**b**; right) on tooth level. Orange circle: mean; bold line: median; box: interquartile range (between 25 and 75%); whiskers: range between 12.5 and 87.5%; grey dots figure the 120 observations; red line: clinically important difference at 3.7 units or 2.7 units

Changes within segments S_1_ and S_2_ from baseline to 3 months after MBA treatment (T_0_ – T_2_) were at worst 1.97 ≈ 2.0 units (ΔE for gingival segment; Table 2), which is less than the threshold of 2.7 units for a clinical meaningful difference (Fig. 6a). Moreover, confidence intervals for the treatment effects in terms of the difference in change indicated no clinically important differences between body and gingival segments (Table 2).

**Table 2:** Treatment effects in terms of the difference in change using linear multilevel models to account for 15 subjects and 120 teeth, and relative treatment effects of the change in terms of the odds ratio of the body segment referred to the gingival segment using ordinal multilevel models

		Linear multilevel model (mixed model)				Ordinal multilevel model	
		Change within gingival segment (S_1_)	Change within body segment (S_2_)	Treatment effect (difference in change)		Relative treatment effect	
	Time points	(95% CI)	Change (95% CI)	Coefficient (95% CI)	*P* value	Odds ratio (95% CI)	*P* value
3D system							
Lightness	T_0_ – T_1_	0.09 (−0.10–0.28)	0.12 (− 0.07–0.31)	0.025 (− 0.11–0.16)	0.716	1.08 (0.62–1.90)	0.780
Lightness	T_1_ – T_2_	0.04 (− 0.15–0.23)	0.02 (− 0.17–0.22)	− 0.017 (− 0.15–0.12)	0.807	0.95 (0.54–1.66)	0.860
Lightness	T_0_ – T_2_	0.13 (0.02–0.25)	0.14 (0.02–0.26)	0.008 (− 0.11–0.12)	0.885	1.05 (0.57–1.94)	0.870
Chroma	T_0_ – T_1_	0.03 (−0.05–0.11)	0.01 (− 0.07–0.09)	− 0.017 (− 0.09–0.06)	0.668	0.87 (0.51–1.49)	0.608
Chroma	T_1_ – T_2_	0.07 (−0.01–0.15)	0.08 (0.004–0.16)	0.017 (− 0.06–0.09)	0.648	1.16 (0.68–1.98)	0.587
Chroma	T_0_ – T_2_	0.10 (0.01–0.19)	0.10 (0.01–0.19)	0.000 (−0.7–0.07)	1.000	0.97 (0.56–1.69)	0.911
L*	T_0_ – T_1_	0.34 (− 0.27–0.94)	0.40 (− 0.20–1.00)	0.066 (−0.41–0.54)	0.786	1.04 (0.65–1.66)	0.879
L*	T_1_ – T_2_	0.03 (− 0.65–0.70)	−0.004 (− 0.68–0.67)	−0.033 (− 0.51–0.44)	0.889	0.83 (0.52–1.33)	0.436
L*	T_0_ – T_2_	0.37 (− 0.001–0.73)	0.40 (0.03–0.77)	0.032 (−0.37–0.43)	0.873	0.92 (0.57–1.47)	0.726
C*_ab_	T_0_ – T_1_	0.19 (− 0.43–0.81)	0.18 (− 0.44–0.80)	−0.008 (− 0.48–0.47)	0.972	1.06 (0.66–1.69)	0.809
C*_ab_	T_1_ – T_2_	0.42 (− 0.13–0.96)	0.29 (− 0.26–0.83)	−0.129 (− 0.55–0.29)	0.543	0.85 (0.53–1.36)	0.498
C*_ab_	T_0_ – T_2_	0.61 (0.19–1.03)	0.47 (0.05–0.89)	−0.138 (− 0.51–0.23)	0.464	0.78 (0.49–1.25)	0.302
ΔE	T_0_ – T_1_	2.10 (1.50–2.69)	2.46 (1.86–3.05)	0.360 (− 0.16–0.89)	0.176	1.43 (0.88–2.33)	0.154
ΔE	T_1_ – T_2_	2.15 (1.64–2.66)	2.35 (1.84–2.86)	0.197 (−0.33–0.72)	0.460	1.23 (0.77–1.97)	0.392
ΔE	T_0_ – T_2_	1.80 (1.39–2.22)	1.86 (1.45–2.28)	0.062 (− 0.37–0.49)	0.777	1.11 (0.68–1.81)	0.688
d(OM1)	T_0_ – T_1_	0.38 (− 0.46–1.22)	0.42 (−0.41–1.26)	0.043 (− 0.56–0.65)	0.889	1.01 (0.63–1.61)	0.979
d(OM1)	T_1_ – T_2_	0.32 (− 0.48–1.12)	0.21 (−0.59–1.01)	−0.11 (− 0.66–0.44)	0.691	0.92 (0.58–1.47)	0.739
d(OM1)	T_0_ – T_2_	0.70 (0.22–1.18)	0.63 (0.15–1.11)	−0.068 (− 0.55–0.41)	0.781	0.96 (0.60–1.54)	0.882
2D system							
L*	T_0_ – T_1_	0.31 (− 0.17–0.80)	0.30 (− 0.19–0.78)	−0.016 (− 0.35–0.32)	0.926	1.01 (0.63–1.60)	0.975
L*	T_1_ – T_2_	0.06 (− 0.37–0.50)	0.05 (− 0.39–0.49)	−0.012 (− 0.31–0.29)	0.936	0.91 (0.57–1.46)	0.704
L*	T_0_ – T_2_	0.38 (− 0.03–0.78)	0.33 (− 0.06–0.75)	−0.028 (− 0.31–0.26)	0.847	0.93 (0.58–1.48)	0.748
C*_ab_	T_0_ – T_1_	0.23 (− 0.29–0.75)	0.17 (− 0.35–0.69)	−0.053 (− 0.36–0.26)	0.738	0.84 (0.53–1.33)	0.449
C*_ab_	T_1_ – T_2_	0.20 (− 0.25–0.66)	0.17 (− 0.28–0.62)	−0.034 (− 0.31–0.24)	0.805	0.90 (0.56–1.44)	0.668
C*_ab_	T_0_ – T_2_	0.43 (0.05–0.82)	0.34 (− 0.04–0.73)	−0.087 (− 0.33–0.15)	0.471	0.77 (0.49–1.23)	0.279
ΔE	T_0_ – T_1_	1.63 (1.17–2.10)	1.74 (1.28–2.21)	0.111 (− 0.26–0.48)	0.552	1.33 (0.83–2.14)	0.239
ΔE	T_1_ – T_2_	1.45 (1.03–1.87)	1.65 (1.22–2.07)	0.199 (−0.16–0.56)	0.271	1.32 (0.82–2.13)	0.260
ΔE	T_0_ – T_2_	1.61 (1.24–1.97)	1.39 (1.03–1.76)	−0.214 (− 0.51–0.08)	0.155	0.80 (0.49–1.28)	0.346

#### 3D-system

At baseline, 13 different shades were measured (Fig. 4b). Four shades with a frequency greater than 30 occurred: 1 M2, 2 L2.5, 2 M3, and 3R2.5 (Fig. 4b). Note that shades 2 L2.5, 2 M3, and 3R2.5 limit a space that is not well covered by the 3D system (Fig. 5; 3R2.5 is nearest neighbor of 3 L2.5). *Chroma* of gingival segments was stronger than that of body segments (*P* = 0.018; Table 1); differences in *lightness* were uncertain (*P* = 0.17; Table 1).

**Fig. 7:**
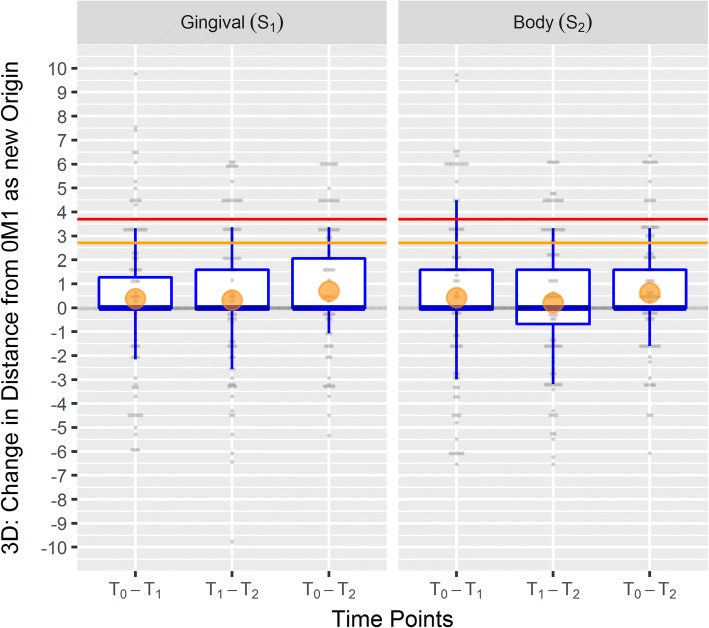
Box plots showing the distribution of the change in distance from 0 M1 for the 3D system on tooth level. Orange circle: mean; bold line: median; box: interquartile range (between 25 and 75 - 50% of the values); whiskers: range between the 12.5 and 87.5% (75% of the values); grey dots figure the 120 observations; change > 0 indicates darker or stronger colors; change <0 indicates lighter or purer colors; red line: clinically important difference at 3.7 units or 2.7 units

Changes within segments S_1_ and S_2_ from baseline to 3 months after MBA treatment (T_0_ – T_2_) were at worst 2.28 ≈ 2.3 units (ΔE for body segment; Table 2), which is less than the threshold of 2.7 units for a clinical meaningful difference. Figs. 6b and 7 illustrate that ΔE is prone to information bias (measurement error). The value of ΔE = 9.9 for T_0_ – T_1_ and T_1_ – T_2_ at the gingival segment as shown in Fig. 6b resulted from a change from 1 M2 to 3 L2.5 and back to 1 M2 for T_0_, T_1_, and T_2_, respectively. This change is more appropriately described in terms of d(0 M1): Values of 9.2, 19.0, and 9.2 for T_0_, T_1_, and T_2_, respectively, correspond to a change in d(0 M1) of 9.8, and − 9.8 for T_0_ – T_1_ and T_1_ – T_2_, respectively, because d(0 M1) allows negative values to describe purer or lighter changes. Moreover, confidence intervals for the treatment effects in terms of the difference in change indicated no clinically important differences between body and gingival segments (Table 2).

## Discussion

During MBA treatment, color changes in bracket (body) and non-bracket (gingival) tooth segments were not clinically relevant. Moreover, body and gingival tooth segments differed in change in tooth color only slightly and possibly by zero. The extent of change in color depended on color metrics (2D, 3D); nevertheless, our findings using different color metrics were sufficiently robust insofar as color change during MBA treatment was not clinically relevant, even if using small thresholds down to 2.3 units for a clinically relevant difference (ΔE).

### Methods of the study

In this study, we preferred electronical measurements instead of visual measurements for several reasons. First, it was assumed that problems due to the regression to the mean [30] which is “one of the most important of all phenomena regarding data and estimation” [31] could not have been substantially reduced by repeated visual measurements; the judger will be biased after the first measurement. Second, we aimed to use measurements of two systems (2D and 3D) for which judgers would have introduced bias regarding the second measurement. Third, four measurements as used internally by the electronic device to compute the overall value increased the reliability according to the Spearman-Brown formula. Fourth, by using quarter points, electronic 2D measurements could have been more accurate than visual 2D measurements. Finally, it could be expected that our adolescent patient group (11–18 years) was homogeneous concerning tooth colors, especially in terms of B color shades of the 2D system. Therefore, it could be assumed that a systematic measurement error will be substantially the same in this highly homogeneous group – an assumption which would not be justified in a sample with a wide age range (and more frequent color shades different from B of the 2D system). This is a crucial point because in presence of a constant systematic measurement error the validity of the measurement of change will not be threatened. In short, we looked for a trade-off between reliability and validity issues, including regression to the mean.

Nevertheless, there are some limitations concerning the electronical measurement methods, including light condition, calibration of the measurement device, reproducibility of the measurements, and visual threshold discussed in the literature [32]. The spectrophotometer Shade Inspector™ was used in our study, because of its good results regarding reproducibility of lightness and chroma found in pilot studies [15, 33]. Other studies, investigating dental color measuring devices did show reliable results as well [34–38].

The Shade Inspector™ is calibrated with a factory-provided selection of industrially fabricated color reference scale (*VITAPAN*® *Classical* and *VITA 3D-Master®*). These color scales originating of different batches were read in and the measurements averaged. Therefore, variations in measurements due to the calibration process are conceivably [39]. The study of *Kohlmeyer* and *Scheller* evaluating VITAPAN® Classical color scale samples, revealed that the individual color scale samples failed to invariably correspond to the respective primary color [40]. In addition, unequivocal findings were reported on color consistency alongst shade guides from the same manufacturer [41, 42]. One in vitro study found that repeatability and accuracy of a dental color measuring instrument (ShadeScan) was influenced by shade guide systems used for testing [43]. In our study, the complete clinical procedure was performed by an experienced orthodontist under standardized conditions (color neutral such as same room, same dental unit and same light conditions by dental unit lamp, patient was covered by a drape, tooth surfaces were always saliva-wet). The electronical measurements were performed by a calibrated examiner [15] in a pilot study. The tooth color measuring device itself operates independently of light on the principle of spectral photometry. However, in a study, evaluating the effect of different illuminants (natural daylight, dental unit lamp, and daylight lamp), the matching repeatability of 2 intraoral spectrophotometers was not completely satisfactory for clinical practice [44]. Therefore, our measurements were taken under standardized conditions as described before. Thus, we do not assume relevant effects by the surrounding light conditions.

Our study has methodological strengths. Notably, two measurements (2D, 3D) at each time point were used, thereby reducing problems due to regression to the mean, which is here the tendency of tooth segment’s colors at the extremes to have less extreme values on subsequent measurements [30]. To reduce the influence of extreme values at the first measurement, it is common to discard the first of three blood pressure measurements of the same examination [45] or to measure the periodontium by the Florida probe thrice given disagreement in first two measurements. Importantly for interpreting of the analysis of change as done herein, the second measurement was performed by the 3D-system, which was considered as the primary outcome. Moreover, we used mixed models as a shrinkage approach and “a way of discounting observed variation that accounts for regression to the mean” [31]. Second, the 2D-system measured at quarter points for the second shade designation number. As the 3D-system did not cover the space of the most frequent 2D shades, the 2D-system added essential information, although limited by the regression to the mean. Third, tooth type as a potentially substantial confounder can only be considered in multilevel analysis. Further, it is not possible to address confounding due to tooth type by the study design. Thus, tooth type cannot be subject of randomizing in a MB study; analysis restricted to the subject level can be misleading. Fourth, we presented not only the original codes of the 2D- and 3D-system but also the transformed values based on the CIE system. As B2 > B1 > B4 > B3 on the L* scale [16]. the shade designation numbers of the original 2D codes cannot be well interpreted. Finally, we used not only ΔE to estimate the treatment effect but also the measure d(0 M1) to allow for purer or lighter changes. In terms of L*, ΔE does not differentiate a lighter change from a darker change given the same ΔE; in terms of C*_ab_, ΔE does not differentiate a purer change from a stronger change. The 3D shade 0 M1 as the new origin of the coordinate system enables us to differentiate lighter/purer changes from darker/stronger changes. 0 M1 as the new origin of the 3D-system is justified for its lightest *lightness* and its purest *chroma*, including the purest red (a*) and the purest yellow (b*). For the 2D- system, no shade has these properties [16].

Unfortunately, there was no sample size calculation for this study. However, we accounted for subject and tooth level to increase statistical power. Moreover, other studies included similar numbers of participants [10, 46]. Besides this limitation, it was not sensitive to adjust for baseline values [47–49], because segments could not be randomized to treatment groups. Therefore, we compared the difference in change from baseline between segments [28, 50, 51].

### Discussion of results

Confidence intervals for the treatment effect for both color systems indicated no clinically important differences between body and gingival segments. Further, changes from baseline to 3 months after MBA treatment (T_0_ – T_2_) were at worst 2.3 units for 3D- system and 2.0 units for 2D-system, respectively, which are less than the threshold of 2.7 units for a clinical meaningful difference.

Previous studies [4, 14] have shown that the enamel color variables are affected by orthodontic bonding and debonding procedures due to tooth cleaning [52], enamel conditioning procedures (etching) [53], and enamel scratches [54]. Other effects, such as staining of enamel and resin material used for the bonding brackets, may also induce color change of teeth during orthodontic treatment. These color change may be the result of demineralization [55], or direct food dye [12, 56]. The staining of the resin material is associated with the color instability of the polymer [57].

Several experimental studies [3, 4, 12–14, 58] investigated the impact of the bonding process on tooth color. Three studies [3, 12, 14] investigating color change after bonding of extracted teeth have not found any indication of a significant influence of the bonding process on tooth color. In another experimental study [13] assessing color changes in bracket areas, significant differences in ∆E were found. Despite the significance of the results, the authors did not consider the color changes visually perceivable for the majority of examiners. *Eliades* et al. [4] reached similar conclusions when examining the influence of different bonding materials. Furthermore, enamel color alterations might also derive from the irreversible penetration of resin into the enamel surface [4]. Moderate evidence exits that shorter resin tags penetration produces less change in enamel color following clean-up procedure and polishing [58]. Self-etching primers produce less resin penetration and these systems produce less iatrogenic color change in enamel following orthodontic treatment [58]. In our study 35%-phosphoric acid was used.

The results of a prospective clinical trial conducted by *Karamouzos* et al. [10] showed significant changes of tooth color (2.1 to 3.6 ∆E units) after orthodontic treatment. The value for the parameter *lightness* (L*) decreased, whereas the values for the parameters a* (value for green-red) and b* (value for blue-yellow) increased. These changes indicated a decrease in tooth *lightness* as well as a change in *hue*, which may be perceptible if a threshold of 1.2 is assumed [26]. In our study, however, we did not find ∆E values greater than 2.7 units, which are considered clinically relevant [26]. Nevertheless, our results are in accordance to a recently published review by *Chen* that there is no strong evidence that orthodontic treatment with fixed appliances alters the original color of enamel [8].

## Conclusion

Within the limitation of this study the MBA treatment can be seen as a safe method with respect to tooth color.
